# Exploring new useful phosphors by combining experiments with machine learning

**DOI:** 10.1080/14686996.2024.2421761

**Published:** 2024-11-07

**Authors:** Takashi Takeda, Yukinori Koyama, Hidekazu Ikeno, Satoru Matsuishi, Naoto Hirosaki

**Affiliations:** a Research Center for Electronic and Optical Materials, National Institute for Materials Science (NIMS), Tsukuba, Japan; b Center for Basic Research on Materials, National Institute for Materials Science (NIMS), Tsukuba, Japan; c Department of Materials Science, Graduate School of Engineering, Osaka Metropolitan University, Sakai, Japan; d Research Center for Materials Nanoarchitectonics, National Institute for Materials Science (NIMS), Tsukuba, Japan

**Keywords:** Phosphor, high-throughput experiment, machine learning, local structure, europium

## Abstract

New phosphors are consistently in demand for advances in solid-state lighting and displays. Conventional trial-and-error exploration experiments for new phosphors require considerable time. If a phosphor host suitable for the target luminescent property can be proposed using computational science, the speed of development of new phosphors will significantly increase, and unexpected/overlooked compositions could be proposed as candidates. As a more practical approach for developing new phosphors with target luminescent properties, we looked at combining experiments with machine learning on the topics of emission wavelength, full width at half maximum (FWHM) of the emission peak, temperature dependence of the emission spectrum (thermal quenching), new phosphors with new chemical composition or crystal structure, and high-throughput experiments.

## IMPACT STATEMENT

The combination of high-throughput experiment and machine learning is a practical approach for developing new phosphor. In this paper, some development examples of new phosphor are presented.

## GRAPHICAL ABSTRACT

**Figure:**
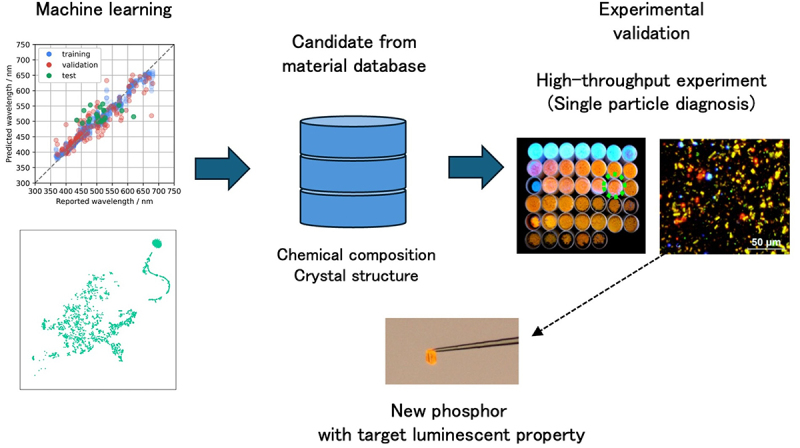

## 1. Introduction

Phosphors are luminescent materials that emit light when excited by external energy (e.g. light, electrons, electric fields, or stress). They have been applied in various fields, such as fluorescent lamps and cathode-ray tube (CRT) displays. Currently, phosphors are used as an essential component of white light-emitting diodes (LEDs). White LEDs have the characteristics of high energy efficiency, compactness, light weight, and long-term stability, and have become widespread as energy-saving lighting and backlights of liquid crystal displays in daily life [1–4]. Among the many types of phosphors (inorganic, organic, and coordination complexes), luminescent center-doped inorganic phosphors are primarily used in white LEDs.

In luminescent center-doped phosphors, a luminescent center is added to a host material. The luminescent center generally occupies the crystallographic site in the host material via solid-solution substitution. Rare earth and transition metal ions are used as the luminescent centers. The rare earth ions Eu^2+^ and Ce^3+^ are the main luminescent centers because their luminescence is highly efficient owing to parity-allowed 4f-5d transitions, and they emit in the visible wavelength range. The broad spatial distribution of the 5d orbitals in the excited states of Eu^2+^ and Ce^3+^ is strongly influenced by the host material, and the luminescence properties are easily changeable. Selection of the host material is one of the most important factors in the development of new phosphors.

**Figure 1.**
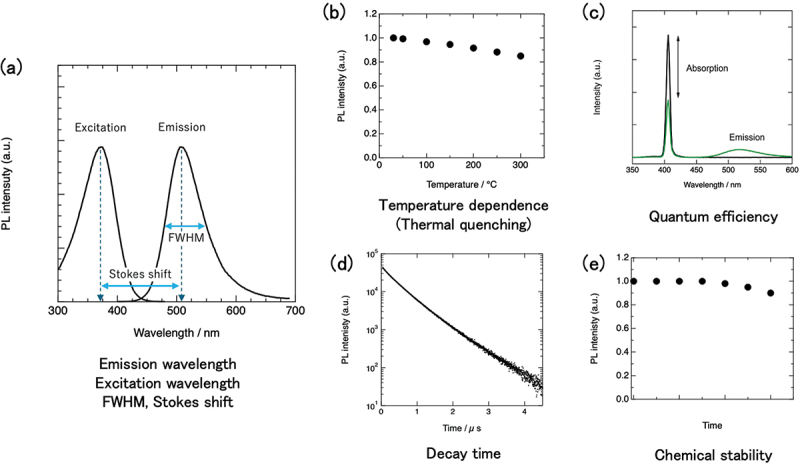
Luminescent properties of phosphor (a) emission wavelength, excitation wavelength, FWHM, Stokes shift, (b) temperature dependence of emission intensity (thermal quenching), (c) quantum efficiency, (d) decay time, (e) chemical stability.

Many luminescent properties should be considered for these applications, as shown in Figure 1 (emission wavelength, excitation wavelength, Stokes shift, full width at half maximum (FWHM) of the emission peak, temperature dependence of the emission spectrum (thermal quenching), quantum efficiency, and decay time). In addition to luminescent properties, in practical applications long-term chemical stability and particle size are important.

In phosphor-converted (pc) white LEDs, phosphors excited by blue LED emit luminescence, and the combination of emission from blue LED and luminescence from the phosphors produces white light. Therefore, blue-excitable phosphors are required. In early pc-white LEDs, only yellow-emitting cerium-doped yttrium aluminum garnet (YAG:Ce) phosphors were used, and the color rendering of white LEDs was poor owing to the lack of a red component. To improve the color rendering, red-emitting nitride phosphors (CaAlSiN_3_:Eu^2+^, Sr_2_Si_5_N_8_:Eu^2+^) have been developed [5,6]. To obtain much higher color rendering, near-UV white LEDs have also been fabricated. Therefore, near-UV-excitable phosphors are necessary. In both cases, multiple broadband-emitting phosphors are used to achieve high color rendering and adjust the color temperature.

By contrast, narrow-band-emitting phosphors are required to match the luminosity curve and enhance the efficiency of white LEDs by reducing invisible light [7,8]. In display applications, narrow-band-emitting phosphors are required to enlarge the color gamut. Thus, based on the emission spectrum, there is demand for a wide variety of emission wavelengths and half-widths.

The luminescence properties depend largely on the host material. For a long time, experimental researchers have selected host materials by trial and error based on their experience and knowledge and found many new phosphors. If a host material suitable for the target luminescence property is proposed based on computational science, the development speed of new phosphors will significantly increase and unexpected/overlooked compositions could be proposed. Although multiconfigurational ab initio calculations of Eu^2+^/Ce^3+^-activated phosphors have been extensively conducted, it takes time to apply this method to the discovery of new phosphors [9–12]. In commercial phosphors, the concentration of the luminescent center is high, and the luminescence properties above room temperature are important. Thus, the calculation conditions become more difficult. Additional approaches are required to achieve faster development of new phosphors.

As a more practical approach for developing new phosphors with target luminescent properties, we looked at combining experiments with machine learning on the topics of emission wavelength, FWHM of emission peaks, temperature dependence of the emission spectrum (thermal quenching), new phosphors with new chemical compositions or new crystal structures, and high-throughput experiments to verify the candidates.

## 2. Emission wavelength

Uitert proposed an empirical formula for emission wavelength, which is the most important luminescence property of phosphors [13]:

(1) $$E=Q\left[1-{\left[V/4\right]}^{1/V}10^{-\left(n\cdot \mathrm{ea}\cdot r\right)/80}\right]$$

where *Q* is the energy level of the lower d-band edge for the free ion, *V* is the valence of the center cation, *n* is the number of coordinated anions, *ea* is the electron affinity of the anion, and *r* is the ionic radius of the host cation substituted by a luminescent center. The empirical formula fits the emission peak data for some Eu^2+^- and Ce^3+^-activated phosphors. However, based on these parameters, host materials with the same cation and coordination number have the same emission wavelength. Dorenbos proposed a detailed formula that introduces additional parameters [14–17]. However, it remains difficult to use this formula to screen for the emission wavelength of a new phosphor.

**Figure 2.**
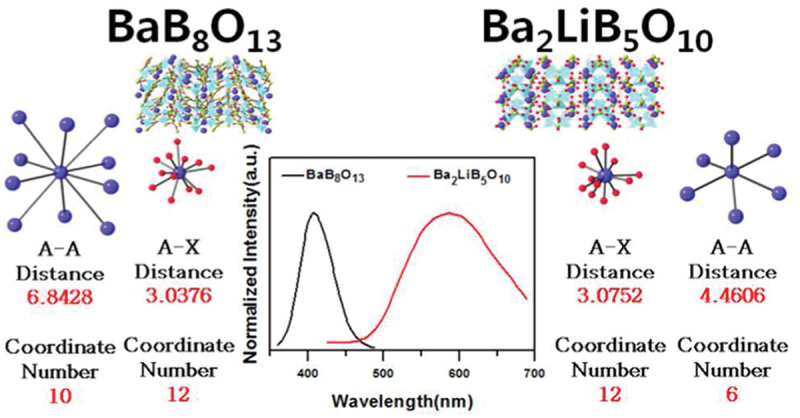
Emission spectra and A-X and A-A local structures for Ba_2_LiB_5_O_10_:Eu^2+^ and BaB_8_O_13_:Eu^2+^. Reproduced with permission from ref. 21, copyright 2015, ACS.

With the development of materials informatics [18–20], applications of phosphor materials have been developed. In a pioneering study, Park et al. used confirmatory factor analysis to predict emission wavelengths [21]. They mined 75 Eu^2+^-activated phosphors with single Eu^2+^ sites in their crystal structures and extracted 32 descriptors. Eight material descriptors were selected to be important through four latent factors (A−X local environment factor, A−A local environment factor, the anion trait factor, and the networking element trait factor, where A is the activator site and X is the anion site). Two borate phosphors, Ba_2_LiB_5_O_10_:Eu^2+^ and BaB_8_O_13_:Eu^2+^, were analyzed using these four latent factors (Figure 2). Although they have similar coordination structures (coordination number and average interatomic distance) for the Ba (Eu) sites, they exhibit widely different emission wavelengths (BaB_8_O_13_:Eu^2+^ 408 nm [22] and Ba_2_LiB_5_O_10_:Eu^2+^ 587 nm [23]). The difference in the emission wavelength is explained by differences in the A-A local environment. Typically, the first coordination sphere is the main factor influencing emission spectra. This suggests that diverse descriptors are necessary to predict emission wavelength.

Nakano et al. proposed a model for predicting the emission wavelength using 241 descriptors related to the chemical composition of host compounds for Eu^2+^-activated phosphors [24]. Prediction models were constructed using a Gaussian process regression (GPR) algorithm with data from 288 Eu^2+^-activated phosphors. The prediction accuracies of mean absolute error (MAE) and root mean squared error (RMSE) were 139 and 189 meV, respectively. The prediction model had a reasonable degree of predictive accuracy only from the chemical compositional descriptors.

Park et al. reported a comprehensive machine learning method for predicting the band gap, excitation energy, and emission energy of Eu^2+^-activated phosphors [25]. They collected 91 Eu^2+^-activated phosphors with a single activator site, extracted 29 descriptors and elemental and structural traits of phosphor hosts, and set up an integrated machine-learning platform consisting of 18 machine learning algorithms. They obtained acceptable holdout dataset test results for peak emission wavelength predictions (R^2^ >0.6 and MSE < 0.02).

**Figure 3.**
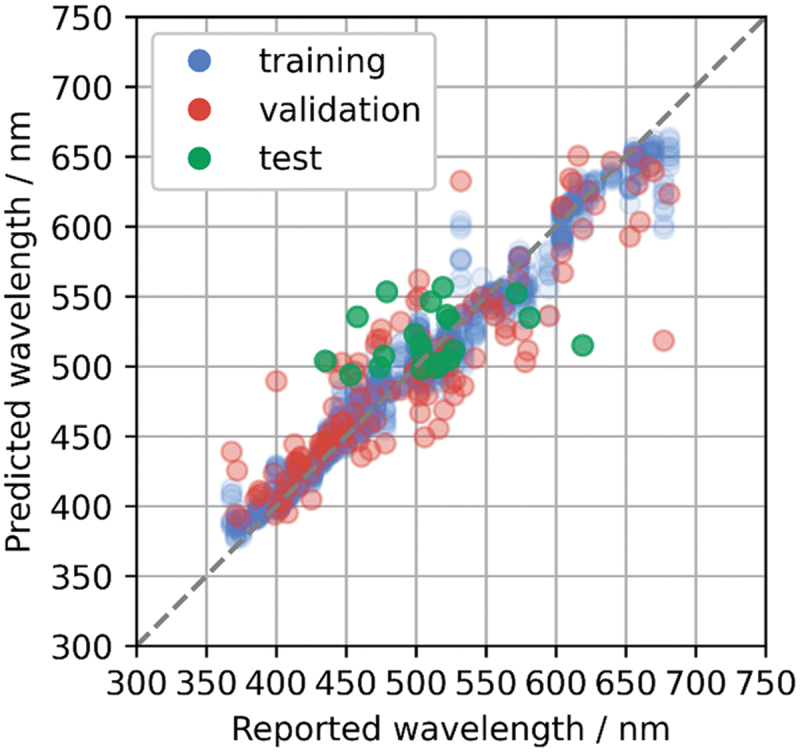
Predicted emission peak wavelengths with respect to reported values for the test data of collected Eu^2+^-activated phosphors (green) using the gradient-boosted regression trees method. The plot is overlaid on the cross-validation results. Reproduced with permission from ref. 26, copyright 2023, RSC.

**Figure 4.**
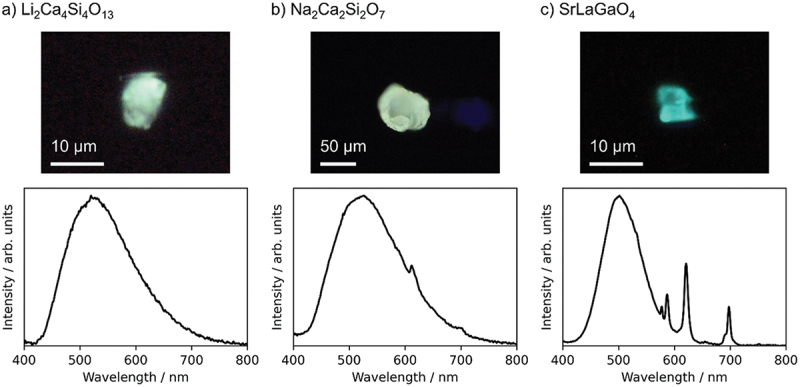
Photo images (upper panels) and emission spectra (lower panels) of particles of Eu-doped (a) Li_2_Ca_4_Si_4_O_13_, (b) Na_2_Ca_2_Si_2_O_7_, and (c) SrLaGaO_4_ under 365 nm LED excitation. Reproduced with permission from ref. 26, copyright 2023, RSC.

The above reports are predictions of emission wavelengths. It has been reported that the constructed prediction model can be used to suggest new phosphors, and candidate materials with targeted properties have been experimentally validated [26]. Koyama et al. constructed an emission peak wavelength model from a dataset of 129 Eu^2+^-activated phosphors obtained from the literature. General-purpose compositional and structural descriptors were used to represent the host compounds of the phosphors. Bootstrap aggregation with the gradient-boosted regression tree method was adopted to obtain high predictive performance and avoid overfitting. The predictive performance of the machine learning model was estimated to be 0.13 eV of MAE and 0.16 eV of RMSE (Figure 3). Using the constructed model, 20 candidate compounds with predicted emission peak wavelengths in the range of 500–550 nm were selected from a material database (AtomWork-Adv) [27]. From the synthesized powders, three new Eu^2+^-activated phosphors, Li_2_Ca_4_Si_4_O_13_:Eu^2+^, Na_2_Ca_2_Si_2_O_7_:Eu^2+^, and SrLaGaO_4_:Eu^2^, were obtained, which successfully exhibited green or blue-green emissions, as designed (Figure 4). Because the powder product was not phase-pure, target composition particles were picked out from the powder, and their crystal structures and emission spectra were characterized (single particle diagnosis), as explained in more detail in section 6.

Despite firing under a reducing atmosphere (5%H_2_/95%N_2_), some products exhibited Eu^3+^ red emission. The valence state of the luminescent center generally depends on the synthesis process, starting materials, and the host structure/composition. Controlling the valence state of the luminescent center is another important factor in the development of new phosphors. The valence state of the luminescent center can be predicted using machine learning.

## 3. Full width at half maximum (FWHM) of emission peaks

Narrow-band-emitting phosphors are required for both lighting and display applications, as described in the introduction. Because the ground state of Ce^3+^ is a doublet (^2^F_5/2_ and^2^F_7/2_), the FWHM of the Ce^3+^ emission peak is wider than that of the Eu^2+^ emission with a singlet ground state (^8^S_7/2_) in the same host material. Eu^2+^-activated phosphors are the main candidates for narrow-band emitting phosphors.

The FWHM of the emission peak was obtained using a configuration coordinate model [28]. For Gaussian line shapes, it is expressed in the following equation:

(2) $$\mathrm{FWHM}\left(T\right)=\sqrt{8\ln2}\overset{ˉ}{h}\omega \sqrt{\mathrm{Scoth}\left(\frac{\overset{ˉ}{h}\omega }{2k_{B}T}\right)}$$

where *S* is the Huang – Rhys factor related to electron-phonon coupling, *ħω* is the mean phonon energy, *k*_*B*_ is the Boltzmann constant, and *T* is the absolute temperature. Although the *S* and *ħω* parameters can be obtained experimentally, it is necessary to synthesize the target phosphor and measure the emission spectra at a low temperature. This is impractical in the exploratory research stage; however, calculating these parameters is difficult. Therefore, an alternative approach is required. The coordination structure of the luminescent center is an important factor that influences the FWHM. When Eu^2+^ occupies a crystallographic site with high symmetry in the host structure, narrow-band emission is observed. A well-known example is the narrow-band red-emitting phosphor SrLiAl_3_N_4_:Eu^2+^ [29]. There are two independent crystallographic sites for Eu^2+^. Although the site symmetry of both sites is ‘1’, both coordination polyhedra are close to cubic. A cuboid local structure around the Eu^2+^ activator is favorable for narrow-band emission. This structural feature is simple and can be extracted from crystal-structure databases. In reality, the local structure of the Eu^2+^ activator is relaxed from the original host structure; however, this was sufficient for screening purposes.

Kim et al. extracted the local structure consisting of a center cation and surrounding anions from the inorganic crystal structure database (ICSD) [30,31]. Materials with cuboid local structures were selected from the extracted local structures. They found 37 cuboid structures that were plausible phosphor candidates. The 37 candidates were categorized by appearance, e.g. ‘Dutch windmill’-shaped structures, edge-sharing-tetrahedron-connected planar cuboid layer structures, edge-sharing-octahedron-connected planar cuboid layer structures, and complex cuboid structures. Because they focused on nitride phosphors, K_2_Zn_6_O_7_ was selected as the starting material owing to its nitride-forming availability. Compositions of Sr_2_[MgAl_5_N_7_]:Eu^2+^ and (Sr,Ca)_2_[MgAl_5_N_7_]:Eu^2+^ were designed. A single phase of Sr_2_[MgAl_5_N_7_]:Eu^2+^ was obtained by powder synthesis in a hot isostatic pressing furnace, and it showed a narrow-band red emission peak at 649 nm with a FWHM of 74 nm, as expected.

**Figure 5.**
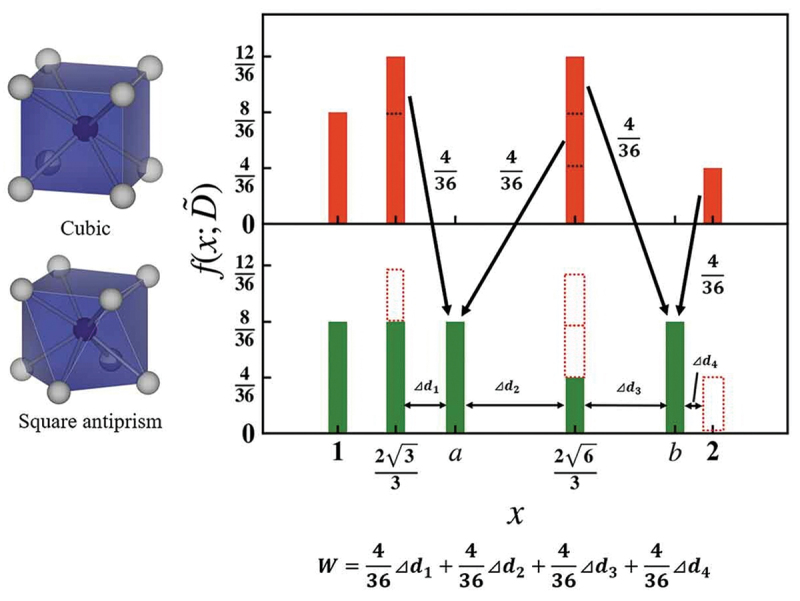
Scheme of the Wasserstein distance between a cubic and square antiprism that distorts the helix angle by 45 °from cubic. Reproduced with permission from ref. 32, copyright 2021, NIMS.

Narrow-band emission was also observed in local structures other than cubic. The reference structure is not limited to cubes and cuboid structures. Other local coordination structures can be used as reference structures for narrow-band emissions. To evaluate the structural similarity between any local structures, Takemura et al. developed a simple and versatile method to obtain quantitative similarity for a local structure around the center ion [32]. The local structure is represented as a distribution of interatomic distances consisting of the center-ligand and ligand-ligand distances. The interatomic distances are normalized by the average center-ligand distance to cancel the difference in the ionic radii. The Wasserstein distance is used to evaluate the dissimilarity between two local structures [33–35]. The Wasserstein distance W, which is based on the transport problem, is the distance between distributions. In data science, the (dis)similarity between data points corresponds to the distance between them. The Wasserstein distance can be calculated using the SciPy package [36]. Figure 5 shows the calculation of the Wasserstein distance between a cubic and square antiprism that distorts the helix angle by 45° from the cubic antiprism, keeping its height as an example. The W distance is calculated as the sum of the products of the transport distance and transport cost; in this case, the W distance was 0.094.

**Figure 6.**
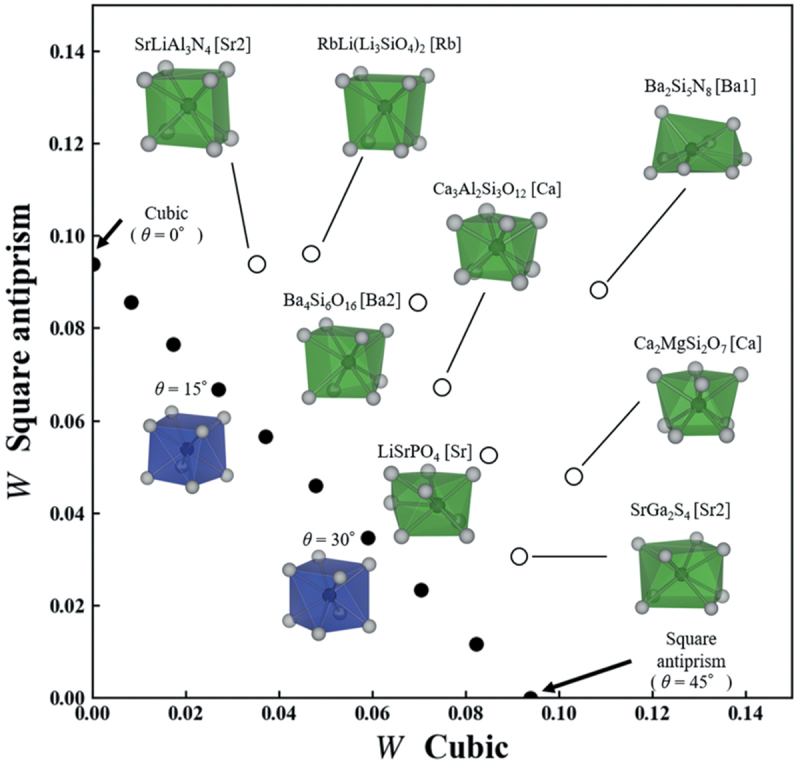
Diagram of the *W* to cubic and square antiprism of eight local structures of known phosphors. Reproduced with permission from ref. 32, copyright 2021, NIMS.

A diagram of the W distance for cubic and square antiprisms of the eight local structures of the known phosphors is shown in Figure 6. Since the coordination number must be determined to extract the local structure from the actual crystal structure, the CrystalNN method [37] can be employed. To distinguish the local structure in the crystal structure, a notation with a central ion in square brackets after the chemical formula, such as CaF_2_[Ca], is used. When there are multiple sites of the same ion, a number is added, e.g. SrLiAl_3_N_4_[Sr1] and SrLiAl_3_N_4_[Sr2]. SrLiAl_3_N_4_[Sr2], which is the furthest on the left side of Figure 6, has the structure most similar to cubic among the eight structures. In contrast, the structure of SrGa_2_S_4_[Sr2], at the bottom of the diagram, is the most similar to square antiprism of the eight structures. Ba_2_Si_5_N_8_ [Ba1], at the top right, is dissimilar to both the cubic and square antiprisms. The dissimilarity using the Wasserstein distance was accurately quantified in the actual local structures. These results agree well with an intuitive approach based on visual appearance.

**Figure 7.**
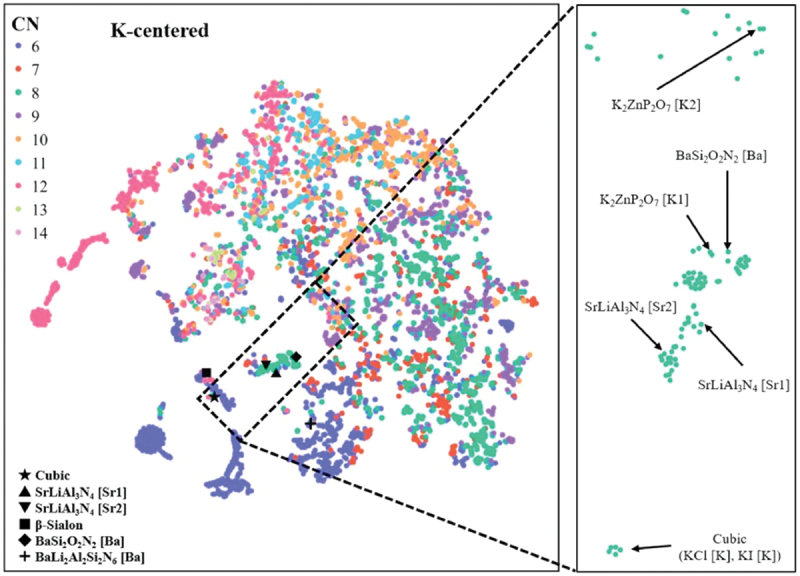
2D t-sne plot of K-centered local structures. Enlarged view indicates only eight-coordinated local structures in the region enclosed by the black dotted line.

Reproduced with permission from ref. 38, copyright 2022, Elsevier.

**Figure 8.**
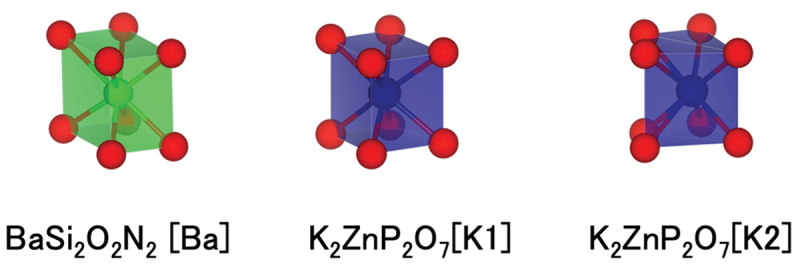
Local structure of Ba site in BaSi_2_O_2_N_2_ and two K sites in K_2_ZnP_2_O_7_.

Narrow-band emitting phosphors were explored using quantitative similarity evaluation by Wasserstein distance and the ICSD crystal structure database [38]. As reference structures with narrow-band emissions, six references of local structures were prepared: ideal cubic local structure, the two Sr-sites of SrLiAl_3_N_4_, the nine-coordinated structure of β-SiAlON [39], the Ba-site in BaSi_2_O_2_N_2_ [40], and the Ba-site in BaLi_2_Al_2_Si_2_N_6_ [41]. The Wasserstein distance was calculated for all pairs of the extracted and reference local structures. To understand the similarity of local structures to reference structures, the distributions of all local structures were visualized as a scatterplot on a 2-dimensional (2D) plane using the t-distributed stochastic neighbor embedding (t-SNE) method [42]. Because the number of extracted local structures is large, they are shown separately according to the center cation. Figure 7 shows a scatterplot of the K-centered local structures. The K sites of simple halides such as KCl are located near the reference ideal cubic structure. Most host crystals with local structures located near the Sr sites of SrLiAl_3_N_4_ contain transition metals, radioactive elements, or toxic elements, and are unsuitable for Eu^2+^-activated phosphors. Other structures near the Sr sites of SrLiAl_3_N_4_ are known phosphors or crystals with cubic-like local structures, as reported by Kim et al. [30]. The K1-site of K_2_ZnP_2_O_7_ is located near the Ba site of the narrow-band-emitting phosphor BaSi_2_O_2_N_2_ in the enlarged plot in Figure 7. K_2_ZnP_2_O_7_ has two K sites, and the K2 site is also near the Ba site in BaSi_2_O_2_N_2_. The local structures of the Ba site in BaSi_2_O_2_N_2_ and the two K sites in K_2_ZnP_2_O_7_ are shown in Figure 8. The band-gap of K_2_ZnP_2_O_7_ is 4.134 eV [43]. It is suitable for phosphors that emit visible light. Therefore, a K_2_ZnP_2_O_7_:Eu^2+^ phosphor was synthesized and its luminescence properties were measured. XRD analysis indicated that the product was a mixture of K_2_ZnP_2_O_7_, KPO_3_, and KZnPO_4_. A single particle of K_2_ZnP_2_O_7_:Eu^2+^ was then selected and analyzed (single-particle diagnosis). The emission spectrum of the K_2_ZnP_2_O_7_:Eu^2+^ particle showed a blue luminescence peak at approximately 440 nm with a narrow FWHM of 30 nm (1549 cm^−1^) [37].

**Figure 9.**
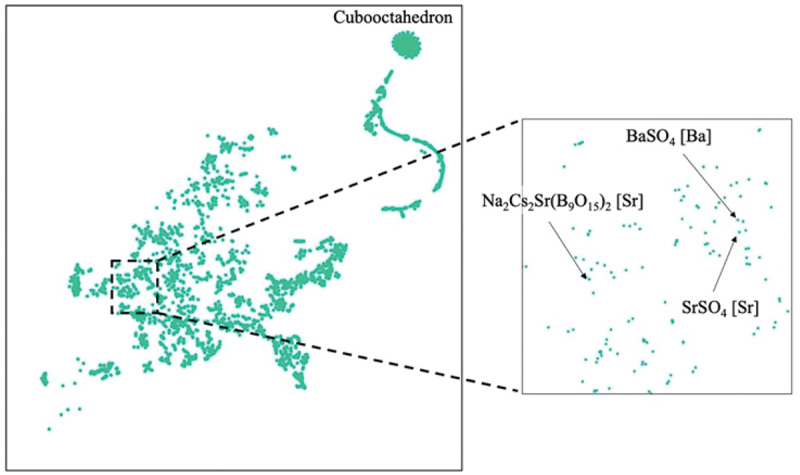
2D t-sne plot of 12 coordinated local structures. Enlarged view indicates the area around BaSO_4_. Reproduced with permission from ref. 44, copyright 2022, ACS.

**Figure 10.**
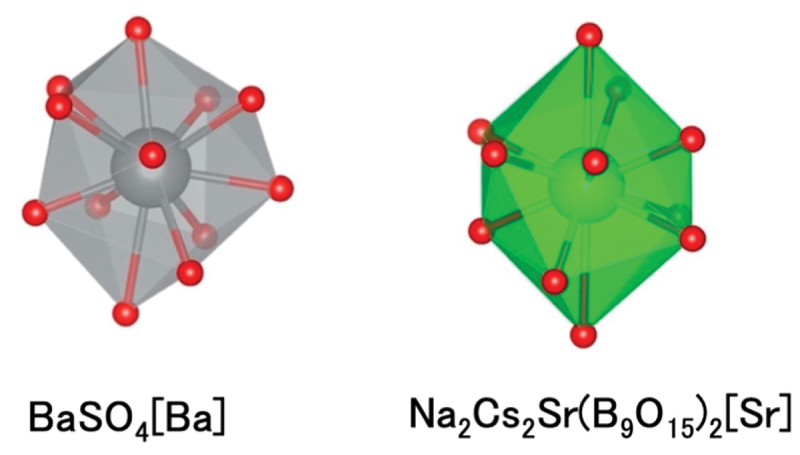
Local structure of Ba site in BaSO_4_ and Sr site in Na_2_Cs_2_Sr(B_9_O_15_)_2_.

The reference local structure of BaSi_2_O_2_N_2_ is flat and rectangular and extends to a cube-like structure. It can be considered a high-symmetry structure. On the other hand, narrow-band emission is observed from distorted local structure and for local structure with different coordination numbers. The same method was applied to the local structure of the sulfate phosphor BaSO_4_:Eu^2+^ (FWHM 1693 cm^−1^). Eu^2+^ occupies the Ba site in the BaSO_4_ crystal structure and is coordinated by 12 oxygen atoms. The Ba – O interatomic distances range from 2.790 to 3.314 Å, and the Ba site has Cs symmetry. The distorted polyhedron is significantly different from a cuboctahedron, which has the highest symmetry among the 12-coordinated structures. Using this distorted local structure as a reference, a search for a similar structure was carried out using the same method [44]. It was found that the Sr site of the Na_2_Cs_2_Sr(B_9_O_15_)_2_ borate is positioned near the Ba site (Figure 9), and powder synthesis of this compound was attempted. In contrast to the characteristic local structures, such as cubes or regular octahedra, it is difficult to visually judge the similarity between the Ba sites in BaSO_4_ and the Sr sites in Na_2_Cs_2_Sr(B_9_O_15_)_2_ (Figure 10). Quantitative similarity evaluation is very useful. A luminescent particle was selected from the powder product mixture and its crystal structure and photoluminescent properties were analyzed. The Na_2_Cs_2_Sr(B_9_O_15_)_2:_:Eu^2+^ particle exhibited an emission spectrum with a peak at 417 nm and a narrow FWHM of 26 nm (1497 cm^−1^).

These studies focused on the coordination structure of the luminescent centers. Clearly, the effect of phonons is important. It is important to consider both coordination structure and phonons using suitable descriptors to expand the range of exploration.

## 4. Temperature dependence of emission spectrum (thermal quenching)

As a phosphor cannot convert absorbed light to emitted light with 100% conversion efficiency and Stokes loss is unavoidable, leading to an increase in temperature of the phosphor under excitation. In white LED applications, the temperature of the phosphor can increase up to 150°C in operation. In laser diode (LD) lighting applications under high-density laser irradiation (>100 W/cm^2^), the phosphor temperature is significantly higher. Although temperature decrease due to heat dissipation can be carried out by complexation of the phosphor with a heat-dissipation agent, it is important that the phosphor can maintain its luminescence intensity at high temperatures.

**Figure 11.**
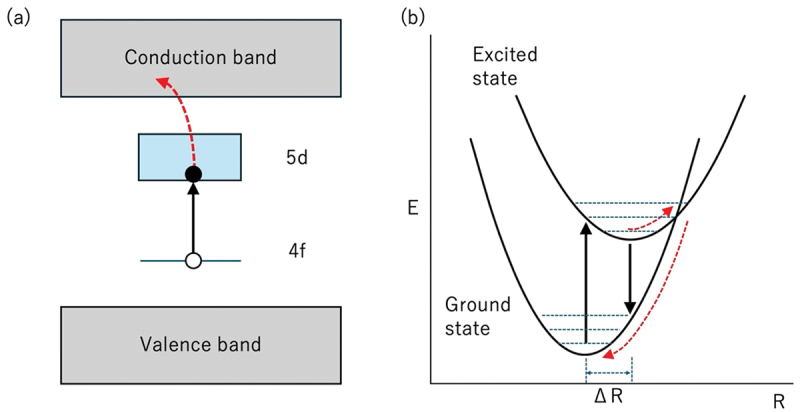
Two mechanisms of thermal quenching, (a) photoionization in the energy diagram, (b) crossover in a configuration coordinate diagram.

The thermal quenching of luminescence can be understood by two mechanisms (photoionization in the energy diagram and crossover in the configuration coordinate diagram, as shown in Figure 11). The excited electrons in the excited state 5d orbitals of Eu^2+^/Ce^3+^ dissipate to the conduction band of the host material if the energy gap between the excited d level and the conduction band is small. This effect is enhanced by increasing temperature. The energy difference between the excited state and the conduction band of the host material is important. In a host material with a large band-gap, photoionization is reduced. The energy position of the d-state in the band-gap is important.

In the configuration coordinate diagram, the electron in the ground-state parabola is excited to the excited-state parabola (up arrow). After relaxation, emission is observed (downward arrow). However, at high temperatures, the excited electrons return to the ground state parabola through the intersection between the excited state parabola and the ground state parabola without emission (red dotted arrow). The nonradiative transition is reduced in the small parabola offset (ΔR) and steep parabola. In a rigid host material, the parabolic offset can be small, the parabolic curvature can be steep, and the crossover point ascends. The Debye temperature is considered a proxy for structural rigidity [45–47].

**Figure 12.**
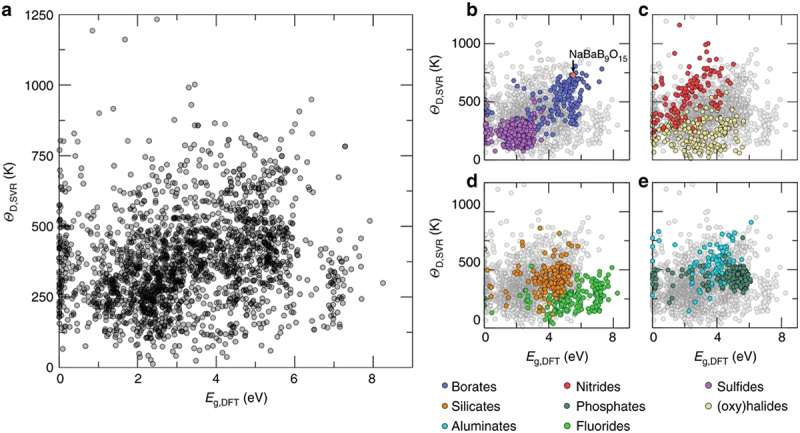
Machine-learning predicted Debye temperature against calculated band-gap. (a) Machine-learning predicted Debye temperature (ΘD, SVR) against the density functional theory calculated band-gap (e.g. DFT) for 2071 compounds. The darker regions occur where there are overlapping data. Classes of common phosphor hosts are highlighted, including (b) borates and sulfides, (c) nitrides and oxyhalides, (d) silicates and fluorides, and (e) aluminates and phosphates. Reproduced with permission from ref. 48, copyright 2018, Springer nature.

Zhuo et al. selected a host material based on the Debye temperature and band-gap of the host material [48]. Because it is difficult to calculate the Debye temperature of many materials, a support vector machine regression model was adopted using a 2610-compound database of material project data [49]. The band-gap was determined using DFT calculations. In the search for host candidates, first, the materials registered in the database were screened based on certain criteria (crystal structure with no disorder or no site mixing for DFT calculations, compounds including elements reported as host materials). The remaining 2071 materials were evaluated using machine learning to predict the Debye temperature and calculate the band-gap, as shown in Figure 12. The distribution varied depending on the type of compound (borates, silicates aluminates, nitrides, phosphates, fluorides, sulfides, or oxyhalides). Among these, NaBaB_9_O_15_ was selected as the host material because of its high Debye temperature and large band-gap. A single-phase powder of NaBaB_9_O_15_:Eu^2+^ was synthesized and exhibited excellent thermal stability. The integrated area of the emission peaks did not change over the entire temperature range of 80–500 K).

From the predicted Debye temperature and calculated band-gap, a thermally robust phosphor was obtained. A prediction model for temperature dependence is also required. However, the amount of reliable temperature-dependence data is poor compared to luminescence spectral data. Further research, including experimental data collection, is necessary to build a prediction model.

## 5. New phosphor with new chemical composition or crystal structure

In the data-driven approach, candidate materials are selected from a material database (e.g. ICSD [30], ICDD [50], AtomWork-Adv [27], PCD Pearson’s Crystal Data [51], or Materials project [49]) using a constructed prediction model and indicator. However, promising candidates from the materials database will run out at some point, even though new materials are regularly added. New host materials not registered in the material database must be used to develop new phosphors with new chemical compositions and crystal structures. One method to obtain new composition materials is ‘multiple substitutions’. Materials registered in the database can be simultaneously substituted with multiple elements. Compared with multiple substitutions of cations, a wide range of materials can be obtained by substituting both cations and anions. Sialon phosphors (Si_6-x_Al_x_O_x_N_8-x_:Eu^2+^, Ca_m/2_Si_12‐m‐n_Al_m+n_O_n_N_16‐n_:Eu^2+^) as practical phosphors for white LEDs are materials obtained by partial multiple substitutions [18,39,52]. In Si_6-x_Al_x_O_x_N_8-x_, Si-N of β-Si_3_N_4_ is replaced by Al-O. In Ca_m/2_Si_12‐m‐n_Al_m+n_O_n_N_16‐n_, Si-N of α-Si_3_N_4_ is replaced by Al-O with introducing Ca.

A representative new phosphor created by ‘multiple substitutions’ is the narrow-band red-emitting phosphor SrLiAl_3_N_4_:Eu^2+^ [29]. The composition SrLiAl_3_N_4_ was obtained by multiple substitutions of UCr_4_C_4_ [53], where U^4+^ and C^4-^ are substituted by Sr^2+^ and N^3-^, respectively. Four Cr^3+^ ions were substituted by one Li^+^ ion and three Al^3+^ion. Its derivative oxynitride and oxide phosphors have been extensively researched as UCr_4_C_4_-type phosphors (i.e. SrLi_2_Al_2_O_2_N_2_:Eu^2+^ and RbKLi_2_[Li_3_SiO_4_]_4_:Eu^2+^) [54,55]. Sr_2_MgAl_5_N_7_:Eu^2+^ was also obtained via multiple substitutions from K_2_Zn_6_O_7_. In both these cases, the original structure before multiple substitutions was selected from cuboid local structures.

**Figure 13.**
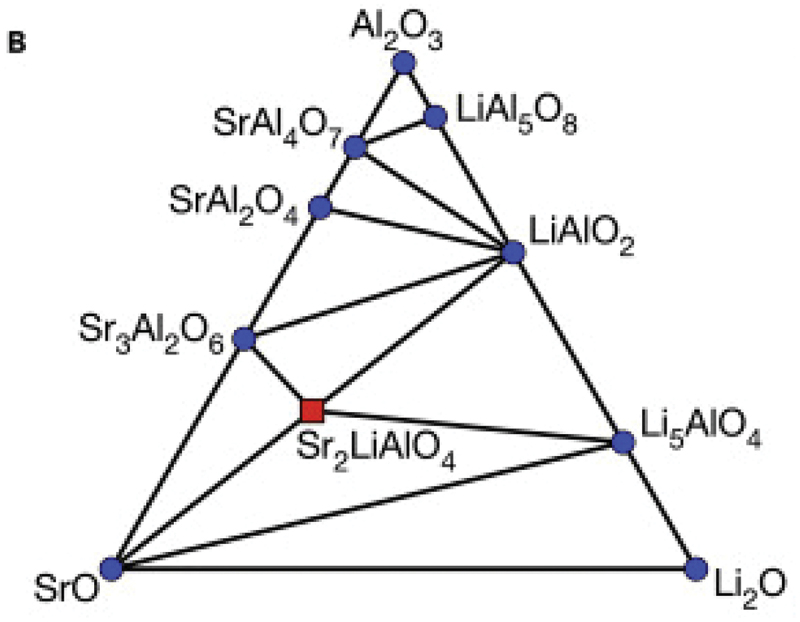
Calculated 0 K SrO-Li_2_O-Al_2_O_3_ phase diagram. Blue circles, known stable phases in the materials project database; red square, new stable quaternary phase, Sr_2_LiAlO_4_. Reproduced with permission from ref. 56, copyright 2018, Elsevier.

Zhenbin et al. focused on quasi-ternary phase diagrams for which compounds have not been reported. They generated 918 new compositions using a data-mined ionic substitution algorithm with seven quasi-ternary phase diagrams: Ba/Sr/Ca-Li-Al-O, Sr-Li-P-O, Ba/Sr-Y-P-O, and Ba-Y-Al-O [56]. In the SrO-Al_2_O_3_-Li_2_O quasi-ternary phase diagram (Figure 13), they discovered Sr_2_LiAlO_4_ from Ba_2_LiReN_4_ by multiple substitutions of Ba^2+^ with Sr^2+^, Re^7+^ with Al^3+^, and N^3-^ with O^2-^. The phase stability and band-gap were evaluated by DFT calculations before synthesis. Eu^2+^-and Ce^3+^-activated phosphors were successfully synthesized by a solid-state reaction. Sr_2_LiAlO_4_:Eu^2+^ showed a green-yellow emission peak at 512 nm, whereas the Sr_2_LiAlO_4_:Ce^3+^ phosphor showed a broad blue emission with a main peak at 434 nm.

**Figure 14.**
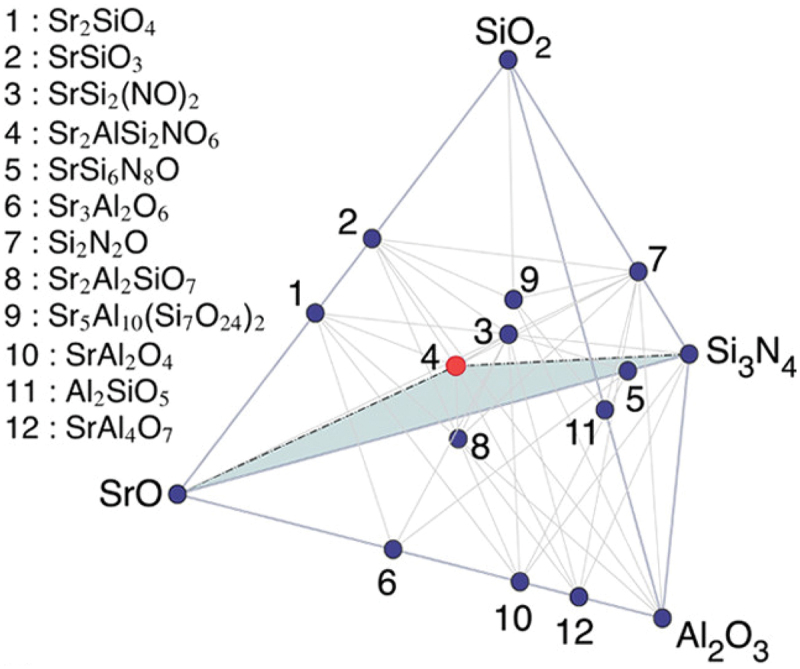
Computation-assisted discovery and structure analysis of Sr_2_AlSi_2_O_6_N. Calculated SrO – SiO_2_–Si_3_N_4_–Al_2_O_3_ three-dimensional phase diagram at 0 K. Dark blue circles, known stable phases in the materials project database; red circle, new predicted phase, Sr_2_AlSi_2_O_6_N. Reproduced with permission from ref. 57, copyright 2019, ACS.

Li et al. discovered a Sr_2_AlSi_2_O_6_N:Eu^2+^ phosphor in a Sr – Al – Si – O – N system (Figure 14). They generated 496 new compositions using a data-mined ionic substitution algorithm and evaluated their stability using DFT calculations. Sr_2_AlSi_2_O_6_N was obtained from Ba_2_ZnGe_2_S_6_O by multiple substitutions of Ba^2+^ with Sr^2+^, Zn^2+^ with Al^3+^, Ge^4+^ with Si^4+^, S^2-^ with O^2-^, and O^2-^ with N^3-^. They synthesized Sr_2_AlSi_2_O_6_N:Eu^2+^ via a solid-state reaction and observed broadband emissions [57]. New phosphors continue to be discovered through multiple substitutions.

The above reports start from the known material and the new phosphor has the same type of crystal structure as the known material. The most difficult challenge is the discovery of a new host material with a new crystal structure. Until now, new materials with new crystal structures have been identified through trial and error by alternating the chemical composition in the phase diagram, discovery by chance in an experiment, or discovery as minerals. Here, an example of the discovery of new nitrides with new crystal structures using machine learning is presented. To enhance the detection probability of a new material, a recommender system for chemically relevant compositions was constructed by machine learning from the ICSD using chemical composition descriptors [58]. The target compounds were ionic compounds in their normal oxidation states. The training dataset consisted of compositions registered in the ICSD as positive cases and compositions not registered in the ICSD as negative cases. Compounds with partial occupancy, unusual oxidation states, and more than 15 atoms in the chemical formula of any constituent element were excluded from the training dataset. The number of positive cases was 33,367. A set of descriptors comprising the means, standard deviations, and covariances of 22 types of elemental representations was used. A random-forest classifier was used in this study. The ensemble size was 10,000. The expected probabilities of positive cases were used as recommendation scores for the compositions.

**Figure 15.**
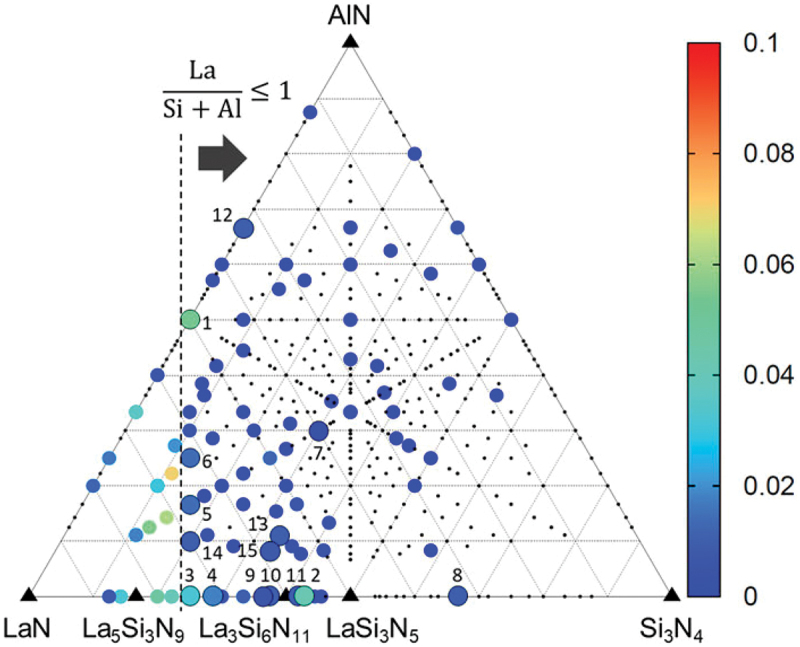
Chemically relevant compositions in the LaN – Si_3_N_4_–AlN pseudo-ternary system. Closed circles correspond to compositions with non-zero recommendation scores as indicated by colors. Large circles indicate top 15 recommended compositions with notations of their ranks. Small dots are compositions whose scores are zero. Black triangles are compositions that are registered in ICSD and used to build the machine-learning model. Reproduced with permission from ref. 58, copyright 2021, AIP publishing.

The recommender system was adopted for the LaN-AlN-Si_3_N_4_ quasi-ternary phase diagram, as shown in Figure 15, and compositions with high scores were experimentally validated. Most of the powder products were mixtures of known phases. In some points, powder XRD analysis identified no known phases, and analysis was carried out using single-particle diagnosis. From point No. 5 in Figure 15, a new compound, La_4_Si_3_AlN_9_, with a new crystal structure was discovered. The composition was the same as the designed composition. Three variants of a known phase were discovered. Although only the crystal structure of the new material is reported in this paper, new phosphors with new structures will be discovered as a result of this type of exploratory research.

## 6. High-throughput experiment

In a validation experiment of the proposed material using machine learning and DFT calculations, powder synthesis was performed according to the proposed chemical compositions. Considering the prediction accuracy, it is reasonable to synthesize a wide range of candidate materials, not only the top candidate material. The synthesis conditions differ depending on the candidate materials used. As the number of candidate materials increases, the number of experiments also becomes enormous and validation experiments takes a lot of time. At least for a candidate materials in the database, its existence is guaranteed. However, the existence of imaginary compositions based on multiple substitutions or recommendation systems is not guaranteed. Even when the energy stability is evaluated by DFT calculations, the synthesis process was not clear. The luminescent properties of a powder are sometimes governed by an impurity phase if the impurity phase is highly luminescent. Therefore, a method to accelerate validation experiments is necessary. This method is a single-particle diagnostic approach [59,60].

**Figure 16.**
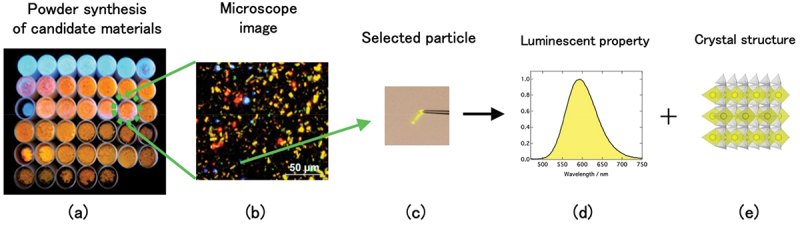
Single particle diagnosis approach. (a) Powder synthesis of candidate materials, (b) optical microscope image of one powder sample, (c) selected particle, (d) luminescent property of the selected particle, (e) crystal structure of the selected particle.

A single-particle diagnostic approach was originally developed to discover new phosphors from a mixture of powdered products (Figure 16). A single-phase powder was obtained by synthesizing a single-phase composition in a phase diagram under suitable synthesis conditions. If the starting composition is a region of mixed phase, the product contains particles of each phase. Owing to non-homogeneous and non-equilibrium conditions in the reaction vessel, there is an increased tendency for phase mixtures. Even if the powder product is a mixed phase, individual particles can be isolated that are single crystals of a single phase. These particles can be treated as candidates for new phosphors. Thus, it is not necessary to obtain a single-phase powder. The single-particle diagnosis approach corresponds to a high-throughput experiment and can accelerate validation experiments. Their crystal structures of selected particles were determined using single-crystal XRD. The luminescence properties (mainly the emission spectrum) were analyzed using a microspectroscopic method. After target phosphors were confirmed, their luminescence properties were measured (excitation spectrum, temperature dependence of the emission spectrum, decay time, and quantum efficiency).

This method focuses on discovering new phosphors. After the discovery of a new phosphor, a scale-up experiment with a high-purity powder sample is necessary to analyze the luminescent properties of the powder sample, optimize the composition (i.e. concentration of the luminescent centers), and produce trial quantities of phosphors.

## 7. Prospects

As a practical approach for developing new phosphors with target luminescent properties that are not explained here, combining experiments and machine learning will continue to be important. There are some points to be noted for the further discovery of new useful phosphors.

Candidate materials with specific luminescent properties have also been proposed. For practical use, it is necessary to simultaneously satisfy several luminescent properties, including emission wavelength, excitation wavelength, FWHM, and temperature dependence. By combining machine-learning models constructed for specific luminescence properties, new phosphors with multiple target properties can be developed.

In a host material with several substitution sites for the luminescent center, an activated phosphor generally exhibits multiple emissions from several sites. These structures are often eliminated in machine learning because of overlapping emission spectra. It is necessary to include these crystal structures in both the learning data and candidates to expand the range of predictions. Complex crystal structures, such as composite crystals, are candidates for host materials.

The data quality should be improved. Reported luminescence properties sometimes differ, even for the same host material. Some luminescence properties depend on the concentration of the luminescent centers. Careful attention is necessary for phosphor selection; however, there are few reports on this topic. It is preferable for researchers to synthesize and measure phosphors; however, this is not an easy task.

Some luminescent properties, such as quantum efficiency (QE), are highly process-dependent because crystallinity has a significant effect on luminescence properties. Although the synthesis process has not yet been opened, QE has been significantly improved by phosphor producers after the discovery of new promising phosphors. Therefore, it is difficult to judge whether the values reported are reliable and specific.

For the validation experiments, it is necessary to synthesize a large number of candidates under various synthetic conditions. More experiments are necessary. Therefore, a high-throughput screening procedure is necessary to accelerate this research. One approach is automated experiments for synthesis and characterization. Automated experiments on powder samples are difficult because of the difficulty in handling powders. However, it is now possible to synthesize powders using robotic inorganic material synthesis laboratories [61]. A second approach is the single-particle diagnostic approach mentioned above. This is useful for identifying target materials and discovering new materials in mixed products. However, the number of dispersed particles is very large even in the small area (Figure 16(b)). The new phosphor particle can be overlooked by visual judgment. Phosphor particles are thoroughly diagnosed by introducing image recognition for the particle search and automated emission measurement [62]. Single crystal XRD with automated sample exchange and sample position adjustment to the X-ray center can speed up the measurement. Automated experiments using a single-particle diagnostic approach will further accelerate the discovery of new phosphors.
